# Facilitating accrual to cancer control and supportive care trials: the clinical research associate perspective

**DOI:** 10.1186/1471-2288-13-154

**Published:** 2013-12-31

**Authors:** David VanHoff, Tanya Hesser, Katherine Patterson Kelly, David Freyer, Susan Stork, Lillian Sung

**Affiliations:** 1 Helen DeVos Children’s Hospital at Spectrum Health, A member of the Grand Rapids Clinical Oncology Program, Grand Rapids, MI, USA; 2 Child Health Evaluative Sciences, Toronto, ON, Canada; 3 Nursing Research and Quality Outcomes, Washington, DC, USA; 4 Center for Cancer and Blood Disorders, Children’s National Medical Center, Washington, DC, USA; 5 Children’s Center for Cancer and Blood Diseases, Children's Hospital Los Angeles, Los Angeles, CA, USA; 6 Blank Cancer and Blood Disorders Center, Blank Children’s Hospital, Des Moines, IA, USA; 7 The Division of Haematology/Oncology, The Hospital for Sick Children, Toronto, ON, Canada

**Keywords:** Cancer control, Supportive care, Clinical research associate, Accrual, Clinical trial

## Abstract

**Background:**

Accrual to Cancer Control and Supportive Care (CCL) studies can be challenging. Our objective was to identify facilitators and perceived barriers to successful Children’s Oncology Group (COG) CCL accrual from the clinical research associate (CRA) perspective.

**Methods:**

A survey was developed that focused on the following features from the institutional perspective: (1) Components of successful accrual; (2) Barriers to accrual; (3) Institutional changes that could enhance accrual; and (4) How COG could facilitate accrual. The survey was distributed to the lead CRA at each COG site with at least 2 CCL accruals within the previous year. The written responses were classified into themes and sub-themes.

**Results:**

57 sites in the United States (n = 52) and Canada (n = 5) were contacted; 34 (60%) responded. The four major themes were: (1) Staff presence and dynamics; (2) Logistics including adequate numbers of eligible patients; (3) Interests and priorities; and (4) Resources. Suggestions for improvement began at the study design/conception stage, and included ongoing training/support and increased reimbursement or credit for successful CCL enrollment.

**Conclusions:**

The comments resulted in suggestions to facilitate CCL trials in the future. Soliciting input from key team members in the clinical trials process is important to maximizing accrual rates.

## Background

Children with cancer have experienced dramatic improvements in survival and about 80% of children are now expected to survive at least 5 years [1]. Much of this improvement can be attributed to the formation of multi-institutional co-operative groups that facilitate conduct of trials for these relatively rare diseases, and the widespread acceptance of clinical trials among pediatric oncologists [2]. Because of this improvement in survival, more attention has been focused on recognition, management and prevention of short- and long-term treatment-related toxicity [3].

Almost all children with cancer in the United States and Canada are treated at institutions affiliated with the Children’s Oncology Group (COG), the largest cancer group focused exclusively on children and adolescents [4]. The COG Cancer Control and Supportive Care (CCL) Committee develops clinical trials focused on the prevention and treatment of acute toxicities in children with cancer [5]. CCL studies are also important in terms of the prevention or minimization of potential late effects of therapy. Outcomes of primary interest have included infection, neurocognition and other neurotoxicities, nutritional status, chemotherapy-induced nausea and vomiting, and quality of life. CCL is supported by the Division of Cancer Prevention at the National Cancer Institute [6].

Timely accrual to studies is important. As is the case with therapeutic studies, CCL trials must be completed in a timely fashion so that the results are relevant and so that patients can benefit from the knowledge derived from these trials. Failure to accrue to these clinical trials results in major financial, resource, scientific and lost opportunity costs. Accrual may be particularly problematic on CCL studies since they tend to be prioritized lower than therapeutic studies by clinicians and institutions [7].

Given the importance of timely accrual and the potential for challenges with the activation of, and accrual to CCL trials [8], we sought to identify modifiable barriers to CCL enrollments in order to develop strategies to improve accrual. While the roles of the physician, institution and patient have been highlighted as important in terms of successful trial conduct [9], others have emphasized the role of institutional clinical trials coordinators or clinical research associates (CRAs) [10]. More specifically, the opinions of CRAs may be particularly important since they are one of the key personnel responsible for accrual to clinical trials. Further, they are one of the only vested individuals who do not have patient care responsibilities and thus, are likely to have a unique and important perspective. Our objective was to identify facilitators and perceived barriers to successful CCL accrual from the CRA perspective.

## Methods

There are approximately 200 institutions that participate in COG clinical trials. A survey was developed by the CCL Committee CRA representative (DV). The survey was distributed to the lead CRA at each COG site with at least 2 COG CCL accruals within the previous year. In the case of non-response, the survey was re-distributed one additional time. The distributions occurred in February and March 2013. Because this survey was developed to improve CCL accrual among COG institutions (in other words, this project was considered a quality improvement project), Institutional Review Board oversight was not required. All CCL trials are performed in compliance with the Helsinki Declaration, and all of the CCL trials described in this manuscript were approved by the National Cancer Institute's Central Institutional Review Board.

The survey consisted of four open-ended questions: (1) What do you feel are the components of successful accrual at your institution? (2) What are the barriers to accrual at your institution? (3) If you could change one thing at your institution to increase accrual to CCL trials what would it be? and (4) How can we (CCL) as a committee contribute to successful accrual at your institution? For question (4), the institutional lead CRA was asked to discuss this issue with his/her principal investigator and to provide a response that reflected that institution’s perspective.

The written responses were collated and categorized using thematic analysis by two investigators (TH and LS) in an iterative fashion [11,12]. Sample quotes were identified to support themes and sub-themes. A third investigator (DV) reviewed the final categorization. The number of comments identified within each them and sub-theme was tabulated.

## Results

Fifty seven COG sites, located in the United States (n = 52) and Canada (n = 5), had enrolled at least 2 participants on CCL studies between December 1, 2011 to November 30, 2012. At the time of survey dissemination, there were 8 CCL studies that were either open or had recently closed to patient accrual within the previous year (Additional file 1: Table S1). The mean number of accruals to CCL studies from the 57 sites was 6.2 (range 2 to 32). The response rate from the 57 sites was 34 (60%).

**Figure 1:**
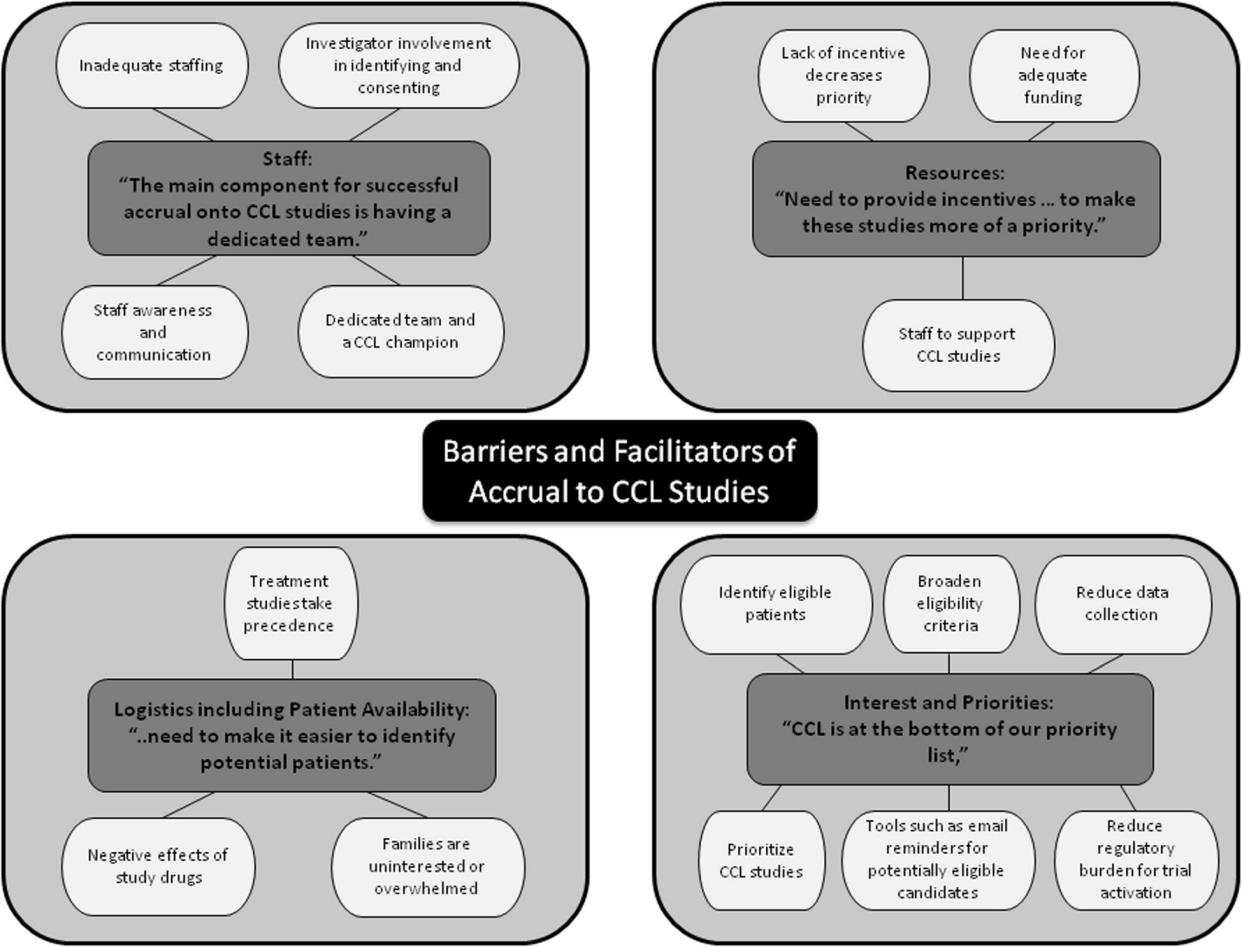
**Barriers and facilitators to accrual to CCL studies.** Illustrates an overview of themes and sub-themes of barriers and facilitators to accrual to CCL studies.

Tables 1 and 2 illustrate the themes, sub-themes and example quotations related to the facilitators and barriers identified at institutions. Comments suggesting what could be done by institutions to improve accrual recapitulated the contents of Tables 1 and 2 and therefore, a separate table is not shown. Table 3 demonstrates suggestions for how COG and the CCL Committee could facilitate CCL accruals. Figure 1 illustrates an overview of themes and sub-themes of barriers and facilitators to accrual to CCL studies.

**Table 1:** Factors related to successful accrual to cancer control studies at institutions

**Themes and sub-themes (number of times mentioned)**	**Example quotations**
**Staff presence and dynamics (35)**	
Dedicated team/staff (11)	"Accrual has proven to be most successful when a CRA/Research Nurse has been highly involved."
Staff awareness of CCL studies (9)	"Awareness (includes CRAs, staff physicians, nurses, fellows) - we feel that very few individuals are aware of these studies." "Weekly COG meetings with research nurse, CRAs and PI; we all know where studies stand, cuts down on emails between each other; helps prioritize studies."
Team communication (6)	"A more clear system of communication when potential patients are identified."
	"It's basically a team approach."
Physician consent/support (6)	"Having a physician's support in identifying and consenting patients is a very important component for accrual."
Presence of a CCL champion (3)	"If we have a specific person who is "championing" the protocol we have more accrual to the protocol."
**Logistics including adequate numbers of eligible patients (7)**	
Ability to identify eligible patients (4)	"Part of successful accrual is being organized in tracking and approaching all eligible patients."
Eligible/willing patients (2)	"Patient willingness."
Timing of approach (1)	"Timing of consent discussion (not at diagnosis as families are overwhelmed with information)."
**Interests and priorities (11)**	
Patient potential benefit (4)	"Protocols that have potential patient benefit or have therapeutic intent (ACCL0933) are prioritized over other CCL studies."
Division/department interest/support (3)	"Our institutional stance is that CCL trials need to be broadly supported and embraced by the COG voting body,"
Prioritization of CCL studies at institution (2)	"Our division as a whole being interested and supportive of COG studies,"
No competing treatments (2)	"If [competing treatments] were to open that could affect the accrual rate for the CCL studies."
**Resources (5)**	
Funding and resources (5)	"CCL trials need to be (…) supported with adequate funding."

Abbreviation: *CCL*, Cancer Control and Supportive Care; *COG*, Children’s Oncology Group; *CRA*, Clinical research associate; *PI*, Principal investigator.

**Table 2:** Barriers to cancer control trial accrual at institutions

**Themes and sub-themes (number of times mentioned)**	**Example quotations**
**Staff presence and dynamics (29)**	
Insufficient staff (16)	"No CRA manpower to dedicate to data collection and study coordination."
Insufficient time (7)	"The most pressing problem is time - not enough time to get all of the protocols through the IRB and not enough to complete data."
	"Sometimes identified patients end up not going on study due to the lack of time for consenting by staff physicians/fellows/NPs in a timely manner."
Lack of communication (4)	"(Need) good communication between the research team and the clinical team."
Lack of awareness (2)	"Lack of knowledge by the clinical staff of available and currently open COG studies."
**Logistics including adequate numbers of eligible patients (26)**	
Eligibility criteria too restrictive (13)	"Apart from inclusion/exclusion, sometimes the need to start within a certain period."
Regulatory barriers to trial activation (6)	"Getting trials open - bureaucratic hoops."
Inability to identify eligible patients (4)	"(…) it is difficult for a CRC to keep on top of all potentially eligible patients,"
Overwhelmed patients/families (3)	"Physicians struggle with when to approach (…) when MDs choose to wait, the studies are often forgotten."
**Interests and priorities (13)**	
CCL not a priority (8)	"(CCL is) at the bottom of our priority list amongst all other front line treatment studies."
	"Investigators focusing all their efforts on the treatment studies."
Lack of family interest (3)	"Feeling like they will be taking another medication that is really not required."
Perceived negative effects of study medications (2)	"Some physicians may be biased about perceived effects/side effects of the study."
**Resources (6)**	
Lack of adequate resources (4)	"Studies that have research funded procedures (are easier)."
Low reimbursement (2)	"Low reimbursement."

Abbreviation: *CCL*, Cancer Control and Supportive Care; *COG*, Children’s Oncology Group; *CRA*, Clinical research associate; *IRB*, Institutional review board; *NP*, Nurse practitioner; *CRC*, Clinical research coordinator; *MD*, Medical doctor.

**Table 3:** Suggested approaches from COG and CCL that would facilitate accrual at the institutions

**Themes and sub-themes (number of times mentioned)**	**Example quotations**
**Staff presence and dynamics (2)**	
Awareness (2)	"Encourage newer COG CRA and RN members to participate."
**Logistics including adequate numbers of eligible patients (14)**	
Ensure study is feasible and minimize data collection (6)	"Simplify, simplify, simplify! Distill CRFs down to the bare minimum data needed to answer the study aims."
Email reminder for eligible patients (5)	"It would be really helpful to have email alerts."
	"Email reminders about patients who have been flagged as potentially eligible can be helpful."
Broaden eligibility (3)	"Keep requirements to a minimum."
	"(…) more CCL trials for disease sites that do not currently have a treatment trial."
**Interests and priorities (2)**	
Patient potential benefit (1)	"Data that shows outcomes of patients enrolled on CCL studies would generate enthusiasm from staff."
Patient incentives (1)	"Patients always like incentives for participation."
**Resources (30)**	
Support from CCL committee (13)	"I think the committee members/protocol staff members are extremely responsive, supportive, and never leave us hanging."
Funding (12)	"Increased per case reimbursement."
	"Have it count as a therapeutic trial for payment/accrual."
Information provision (4)	"CCL study sessions at COG Fall Meeting (…) was extremely helpful to learn from what worked and what didn't work at other sites. It demystified a lot of my apprehensions in opening the study at my institution."
	"Have a meeting with all the lead CRAs and review protocols that are available and ask for input."
Centralized CCL staff (1)	"Provide a CCL centralized staff that do the regulatory for CCL protocols remotely and abstract and submit the data either remotely or otherwise."

Abbreviation: *CCL*, Cancer Control and Supportive Care; *COG*, Children’s Oncology Group; *CRA*, Clinical research associate; *RN*, Registered nurse; *CRF*, Case report forms.

In general, there were four major themes in each of the areas: (1) Staff presence and dynamics; (2) Logistics including adequate numbers of eligible patients; (3) Institutional interests and priorities; and (4) Resources. Facilitators and barriers within each theme tended to identify the same issue from different perspectives. For example, under “staff presence and dynamics”, the main facilitators were presence of a CCL champion, a dedicated team for CCL studies, and communication and awareness of CCL studies within that team. Involvement of physicians was highlighted as particularly helpful, especially in regard to obtaining consent for some types of studies such as studies requiring an investigational new drug application. Conversely, lack of a team approach, limited time, lack of communication and lack of awareness were identified as major barriers to patient enrollment. More specifically, lack of awareness focused on the lack of knowledge about open CCL trials and which patients would be eligible for these trials.

"If we have a specific person who is "championing" the protocol we have more accrual to the protocol."

“Logistics” focused on whether there were adequate numbers of eligible patients available; CRAs highlighted the importance of wide or non-restrictive eligibility criteria. In terms of study design, CRAs stressed the need for studies that are feasible and practical with minimal data collection requirements. CRAs reported that enrollment onto CCL studies may be enhanced where other competing studies can be minimized, such as after the initial period of diagnosis and when there are fewer disease-specific therapeutic studies available.

“Timing of consent discussion (not at diagnosis as families are overwhelmed with information)."

In terms of “interests and priorities”, a preference was indicated for interventional studies with potential therapeutic benefit for patients, over those that are purely descriptive. In contrast, concern about side effects of CCL pharmaceutical interventions was raised. The possibility for monetary patient incentives was also mentioned.

“Protocols that have potential patient benefit or have therapeutic intent (ACCL0933) are prioritized over other CCL studies."

A large number of comments were generated in terms of “resources”. On multiple occasions, greater per-case reimbursement and reimbursement for study procedures were emphasized. A request for central CRA resources to manage some of the regulatory requirements was identified. In addition, a suggestion that CCL accruals “count” similar to therapeutic accruals in terms of institutional stance within COG was made. Finally, the importance of information provision and ongoing education and training was noted.

"CCL trials need to be (…) supported with adequate funding."

## Discussion

We identified that four themes important for patient accrual on COG CCL studies are staff, logistics, interests/priorities and resources. The CRAs generated concrete and reasonable suggestions to facilitate accrual to CCL trials in the future. Such approaches begin at the study design/conception stage and include ongoing training/support and increased reimbursement or credit for successful CCL enrollment.

An emerging body of evidence suggests that the CRA role is important to successful trial implementation and accrual [10]. These tasks include, but are not limited to identification and enrollment of patients, collection of samples and outcome data, tracking questionnaires, submitting adverse events, providing education to participants, and educating healthcare professionals on trial conduct [13]. Identification of individuals responsible for such tasks can improve enrolment rates considerably [14]. The role of the CRA is particularly important since clinicians are typically already working at maximal capacity and cannot take on the extra administrative and task burdens of clinical trial activities [8].

Survey responses suggest that staff presence and dynamics are key elements for institutional success in conducting CCL research. Teams appear to be typically composed of physicians, nurses, CRAs, pharmacists and other healthcare professionals. As a way to address this issue, we have proposed workshops where successful institutions that have created “CCL teams” present their systems to the membership and serve as role models and resources for other institutions. For example, the COG CCL Committee organized a well-attended session at the Fall 2012 COG meeting and plan to continue to hold similar sessions.

We also found that involvement of the physician appears to be important, at least for some study types. Heiney et al. also identified that some studies rely upon physician support depending on trial type [8]. The identification of a CCL champion was also highlighted as an important issue. We define a CCL champion as a member of the pediatric oncology clinical research team (may be a physician, nurse, CRA or other team member) who has a strong interest in, and commitment to CCL research. He/she carries out several important functions including ensuring CCL studies are activated by the institution in a timely fashion, developing systems for identifying and screening potential subjects, and educating the patient, family and entire healthcare team about CCL protocols. We have begun to address this issue in a more formal manner by proposing the concept of a CCL responsible individual for each site. This individual would then act as the CCL champion and could represent the institution at meetings and when institutions are polled for their enthusiasm regarding a potential CCL study. We believe that identification of institutional CCL champions is a particularly important step toward enhancing accrual rates.

The CRAs in our sample noted that greater per-case reimbursement and more “credit” for CCL enrollments would be beneficial. In COG, therapeutic and CCL enrollments tend to provide the same reimbursement value. However, funding to travel to the annual meeting is currently calculated based on therapeutic enrollments and CCL enrollments are not considered. If CCL enrollments are to be targeted, both therapeutic and CCL trials should be considered in any funding formula.

The CRAs also suggested a central CRA who can take over some of the regulatory burdens from sites. Regulatory burdens appear to be increasing in spite of the presence of the Central Institutional Review Board. O’Mara et al. has previously described challenges to accrual for NIH-funded research in the palliative care area and noted that regulatory hurdles and patient accruals were important limitations even among well-funded studies [15]. Carter and colleagues also stressed the importance of a central coordinator center to facilitate study activation at individual centers and to problem-solve issues quickly and efficiently [7].

The strengths of our study include soliciting the opinions of the CRA membership, a group critical to co-operative group trial conduct. This approach is novel and these opinions are not well known. Further, we obtained opinions from many institutions; this approach increases the generalizability of our findings. However, our results must be interpreted in light of its limitations. We did not survey sites with no CCL accruals; these are institutions that may be facing the greatest challenges to CCL accrual and may have unique issues. Second, while we believe these comments are generalizable to non-CCL non-therapeutic studies such as epidemiology and survivorship studies, generalizability to therapeutic trials or outside of the pediatric setting is not assured. Finally, as a descriptive study based on opinion only, the strength of evidence is limited in our report.

Future research should focus on surveying institutions who do not participate in CCL trials as these responses will be particularly informative. The ideal format for surveying these sites may differ and more specifically, telephone contact may yield better response rates since non-participation may reflect lack of interest in CCL studies. Future studies may also consider a mixed method design or include additional qualitative assessment protocols to explore further the issue of non-participation.

## Conclusions

In summary, this study resulted in suggestions to facilitate CCL trials in the future. Many suggestions were actionable including study designs which are feasible and which avoid the initial diagnostic period, promotion of CCL teams through role modeling, frequent CRA education and lobbying to improve institutional credit for each CCL enrollment. Some suggestions will be more difficult to implement such as a central regulatory personnel. Soliciting input from key team members in the clinical trials process is important to maximizing accrual rates.

## Abbreviations

CCL: Cancer control and supportive care; COG: Children’s Oncology Group; CRA: Clinical Research Associate.

## Competing interests

The authors declare that they have no competing interests.

## Authors’ contributions

DV developed and distributed the survey. LS and TH collated and categorized written responses, and DV reviewed final categorization. LS and DV were responsible for writing the manuscript. TH, KK, DF and SS contributed to data collection and revision of the manuscript. All authors read and approved the final manuscript.

## Pre-publication history

The pre-publication history for this paper can be accessed here:

http://www.biomedcentral.com/1471-2288/13/154/prepub

## Supplementary Material

Additional file 1: Table S1 Cancer control and supportive care studies open or recently completed at the time of survey dissemination. Click here for file
