# Supplementary material for: Facilitating accrual to cancer control and supportive care trials: the clinical research associate perspective
Source: BMC Med Res Methodol. 2013 Dec 31;13:154. doi: 10.1186/1471-2288-13-154 (PMC3897981; doi:10.1186/1471-2288-13-154)
Supplement: Additional file 1: Table S1 — Cancer control and supportive care studies open or recently completed at the time of survey dissemination. [file 1471-2288-13-154-S1.doc]

**Additional file 1: Table S1. Cancer control and supportive care studies open or recently completed at the time of survey dissemination**

| **COG ID** | **Abbreviated Title** | **Design** | **Primary Aims** | **Sample Size** |
| --- | --- | --- | --- | --- |
| ACCL0431 | Phase III RCT of Sodium Thiosulfate to Prevent Cisplatin-Induced Ototoxicity | RCT | To evaluate whether sodium thiosulfate is an effective and safe means of preventing hearing loss in children receiving Cisplatin chemotherapy. | 135 |
| ACCL05C1 | Prospective Study of Ototoxicity in Children Receiving Cisplatin | RCT | To determine the optimal criteria for determination of ototoxicity. | 282 |
| ACCL0731 | RCT of Glutamic Acid to Decrease Vincristine Toxicity | DBRCT | To determine if patients treated with l-glutamic acid hydrochloride in conjunction with a vincristine-containing treatment regimen have a decrease in neurotoxicity as compared to those in the placebo group. | 250 |
| ACCL0922 | Phase II RCT of Modafinil for Neurocognitive Deficits | DBRCT | To determine whether modafinil, compared to placebo, is associated with improvement in neurocognitive function in children with cognitive impairment after treatment for a primary brain tumor. | 228 |
| ACCL0933 | RCT of Caspofungin to Prevent Fungal Infection in AML | RCT | To determine if prophylaxis with caspofungin administered during periods of neutropenia following chemotherapy for AML is associated with a lower incidence of proven or probable invasive fungal infection compared with fluconazole. | 550 |
| ACCL0934 | RCT of Levofloxacin to Prevent Bacterial Infection | RCT | To determine if levofloxacin given prophylactically during periods of neutropenia to patients being treated with chemotherapy for acute leukemia or undergoing HSCT will decrease the incidence of bacteremia. | 532 |
| ACCL1031 | RCT of Caphasol to Prevent Mucositis | DBRCT | To determine if topically administered Caphosol, rinsed orally four times daily at the initiation of conditioning for HSCT, reduces oral mucositis as demonstrated by a decrease in duration of severe oral mucositis compared to placebo. | 200 |
| ACCL1033 | A Comprehensive Approach to Improve Medication Adherence in Pediatric ALL | RCT | To test the feasibility, utility, and efficacy of a technologically sophisticated, web-based medication scheduling and text-messaging reminder system coupled with a multimedia-based interactive patient education program to increase medication adherence in children with ALL at high risk for non-adherence. | 570 |

Abbreviations: HSCT – hematopoietic stem cell transplantation; RCT – randomized controlled trial; DBRCT – double-blind randomized controlled trial; ALL- acute lymphoblastic leukemia; AML – acute myeloid leukemia
